# Existential Therapy for Children: Impact of a Philosophy for Children Intervention on Positive and Negative Indicators of Mental Health in Elementary School Children

**DOI:** 10.3390/ijerph182312332

**Published:** 2021-11-24

**Authors:** Catherine Malboeuf-Hurtubise, Carina Di Tomaso, David Lefrançois, Geneviève A. Mageau, Geneviève Taylor, Marc-André Éthier, Mathieu Gagnon, Terra Léger-Goodes

**Affiliations:** 1 Department of Psychology, Bishop’s University, Sherbrooke, QC J1M 1Z7, Canada; 2 Department of Psychology, Université de Montréal, Montréal, QC H3T 1J4, Canada; carinaditomaso@hotmail.com (C.D.T.); g.mageau@umontreal.ca (G.A.M.); 3 Department of Educational Sciences, Université du Québec en Outaouais, Saint-Jérôme, QC J7Z 0B7, Canada; david.lefrancois@uqo.ca; 4 Department of Education and Pedagogy, Université du Québec à Montréal, Montréal, QC H2L 2C4, Canada; taylor.genevieve@uqam.ca; 5 Department of Didactics, Université de Montréal, Montréal, QC H3T 1J4, Canada; marc.andre.ethier@umontreal.ca; 6 Department of Education, Preschool and Primary School Teaching, Université de Sherbrooke, Sherbrooke, QC J1K 2R1, Canada; Mathieu.Gagnon3@USherbrooke.ca; 7 Department of Medicine and Health Sciences, Université de Sherbrooke, Sherbrooke, QC J1K 2R1, Canada; terra.leger-goodes@usherbrooke.ca

**Keywords:** existential therapy, philosophy for children, self-determination theory, school-based intervention

## Abstract

Background: Philosophy for children (P4C) was initially developed in the 1970s and served as an educational program to promote critical thinking, caring, creative reasoning and inquiry in the educational environment. Quasi-experimental research on P4C, a school-based approach that aims to develop children’s capacity to think by and for themselves, has suggested it could be an interesting intervention to foster greater basic psychological need satisfaction in children in school settings. Objective: The goal of the present study was to evaluate the impact of P4C on basic psychological need satisfaction and mental health in elementary school students. Method: Students from grades one to three (*N* = 57) took part in this study and completed pre- and post-intervention questionnaires. A randomized cluster trial with a wait-list control group was implemented to compare the effects of P4C on students’ mental health. Results: Analyses of covariance (ANCOVAs) revealed a significant effect of group condition on levels of autonomy and anxiety, after controlling for baseline levels. Participants in the experimental group showed higher scores in autonomy, when compared to participants in the control group, and participants in the experimental group showed lower anxiety scores, when compared to participants in the control group. Conclusion: Overall, results from this study show that P4C may be a promising intervention to foster greater autonomy in elementary school children, while also improving mental health.

## 1. Introduction

In North America, the prevalence of psychological disorders in youth has risen over the past decade [1]. Major contributors directly affecting this prevalence are the increase in diagnoses, along with the lack of accessibility and affordability of mental health care [2]. Moreover, only one in five children under the age of 12 receives adequate treatment for mental illness [3]. As such, it has been recommended that empirical, evidence-based prevention strategies are developed and implemented in school settings to help foster greater well-being in youth and to render mental health services accessible to a larger number of children [4].

Fostering greater mental health, well-being and overall resiliency to stress has been strongly emphasized in both the American and Canadian elementary school systems [5,6]. Issues pertaining to psychological health and its impacts on academic success and perseverance are of central importance to all school actors and to youth’s parents alike. Decades of research in psychology, education and philosophy have shown multiple benefits that come from higher basic psychological need satisfaction in children, namely with regards to overall mental health, well-being and academic success [7]. Teachers’ interventions that aim to increase basic psychological need satisfaction have also shown promise in fostering better mental health in children in school settings [8,9,10,11].

In recent years, the concept of mental health has been best understood as comprised of both positive (e.g., basic psychological needs satisfaction and well-being) and negative indicators (i.e., symptoms of psychopathology such as anxiety, depression and inattention) [12]. In light of this distinction, in Canada, preventive strategies aiming to foster positive indicators of mental health in youth have gained in influence over the past years, namely because of such programs’ ability to prevent negative indicators of mental health from arising [13]. Given the fact that children spend the majority of their time in a school setting, school-based programs have been suggested as a promising option to promote well-being and increase basic psychological needs satisfaction, while preventing the development of negative indicators of mental health such as anxiety and depression in youth [14]. Namely, philosophy for children (P4C), a school-based approach that aims to develop children’s capacity to think by and for themselves, has shown promise in its ability to positively influence well-being and to prevent anxiety and depression [15,16,17].

### 1.1. Philosophy for Children (P4C)

P4C was initially developed by Lipman in the 1970s, and can be defined as an educational program to promote critical thinking, caring, creative reasoning and inquiry in the educational environment [16,18]. The initial aim of P4C was to help students become reflective and critical thinkers, to gain mastery over their learning environment and thus to become more self-determined [19]. As a result, the majority of the P4C literature to date focuses on validating P4C’s effectiveness with respect to academic and cognitive performance, although some have researched the impact of P4C on empathy, emotions and creativity [20,21]. Indeed, a recent meta-analysis assessing P4C’s effect on youth’s cognitive ability has suggested that P4C has a moderate, yet positive, overall effect on both elementary and high school students’ learning and reasoning abilities [20].

Although the cognitive benefits of P4C have been repeatedly empirically supported, literature addressing the positive psychological outcomes of P4C remains very limited [15]. A handful of quasi-experimental and correlational studies analyzing the psychological outcomes of P4C have emerged with time. These studies have shown, overall, that P4C has a positive effect on psychological outcomes such as self-efficacy, critical openness and relative skepticism [17], but also self-esteem [22,23,24], resilience and happiness [15]. Despite the growing evidence of P4C’s impact on non-cognitive properties, the available literature remains quasi-experimental and thus lacks rigor to provide solid and valid evidence-based data supporting its implementation in school settings to foster greater well-being in children. As such, one aim of the present study was to evaluate whether a P4C intervention could be effective in fostering greater well-being in elementary school children using a randomized cluster trial.

Typically, P4C is delivered in a classroom setting and is facilitated by an educator (e.g., teacher, counsellor or school psychologist). During this time, students are presented with a philosophical primer, which can take multiple forms, such as a short story, a comic strip or a video clip introducing the philosophical theme to be discussed [25]. Primers act to stimulate discussion—or philosophical dialogue—between children [16]. Speech through dialogue is central to the practice of P4C, because it forms a “philosophical community of inquiry” [18], which originates as a scientific or philosophical concept from major American philosophers associated with the pragmatist school developed by Peirce or Dewey, among others [26]. As P4C revisits the pragmatic approach to philosophy, it is a practice of philosophy that engages children in a process of philosophical investigations. This is achieved through cognitive, social and affective means where the content of the discussion is not as important as the process; the only constraints of P4C are related to the form of the discussion itself [27]. This process serves as a safe environment where children can learn to question, reflect on problems, reason, conceptualize, rationalize their own thoughts, interrogate assumptions and work with ambiguities [16,28,29]. During P4C activities, the educator’s role is to ask open-ended questions to stimulate dialogue and provide a supportive emotional climate for the students to explore different existential issues [30]. P4C thus provides an environment from which children can learn how to make inferences about their experiences, which is “fundamental to a child’s ability to acquire meaning” [18]. Not only does P4C allow children to expand their skills to develop their thoughts through dialogue, but it also teaches them to explore their own ethics relating to values and norms. This aligns with the Quebec school institutions’ values that aim to help children become competent, creative, responsible and fully engaged citizens [31]. Further, the Quebec Ministry of Education also conveys that the overreaching goal of education is to assist students in becoming citizens who participate actively in their society [32]. The epistemological context of P4C thus creates a social environment of inquiry that favors this development [33]. Similarly, P4C aims to help children think by and for themselves with the intention of developing their critical thinking skills [27].

As such, P4C relies on similar principles as existential therapy, which in turn may shed light on the psychological processes that are facilitated by P4C. The theoretical basis of P4C relates to existential therapy and self-determination theory, which are presented in the next sections.

### 1.2. P4C and Existential Therapy

Existential psychology is a branch of psychology which places emphasis on the phenomenological experience of the individual [34], and studies how they come to terms with life’s essential dilemmas and the condition of human existence [35]. The existential therapeutic approach focuses on the subjective experience of the individual and their existential anxieties [34]. In the form of a therapeutic dialogue, the goal of existential therapy is to enable clients, by emphasizing their strengths and encouraging them to realize their full potential [35]. Existential therapy has been developed and empirically supported for adults only. To our knowledge, it has not been adapted for children, or for a larger scale use in school and group settings with youth.

Similar to adults, children strive to allocate meaning to their lives and make sense of the world around them [36]. Although the main focus of P4C is more universal and less centered on the individual (as opposed to the therapy process), themes such as identity, time, being and death are discussed in P4C activities, and are simultaneously main ideas addressed throughout existential therapy [34,37]. Given the similarity between P4C and existential therapy, it is likely that P4C may have similar positive effects.

Existential therapy also offers information on the psychological processes that may account for its positive effect. In particular, it suggests that increasing people’s awareness of self and autonomy is important in making sense of the world (i.e., meaning making), and, in turn, decreasing psychological distress and psychopathology, and increasing overall well-being [38,39,40]. It relates to P4C as it is thought to provide a dialogued environment in which children are empowered by the responsibility to participate in discussions, become aware and reflect on their own thoughts, priorities and values, which, in turn, is hypothesized to foster greater autonomy and well-being [41]. The aim to expand an individual’s awareness of their strengths and potential for growth [42] is also at the core of self-determination theory (SDT) [43]. This theory offers evidence for the importance of basic psychological need satisfaction, and particularly the need for autonomy.

### 1.3. Existential Therapy and Self-Determination Theory

SDT is an empirically based metatheoretical approach to human motivation, development and well-being. According to SDT, the satisfaction of people’s innate psychological need for autonomy (a sense of volition and self-endorsement of one’s actions), along with the needs for relatedness (a sense of belonging) and competence (a sense that one has an effective impact on one’s environment) is at the core of optimal development and functioning [7]. In fact, the need to act volitionally has consistently been positively related to psychological well-being and self-esteem in youth and adults alike in a variety of settings, namely in elementary schools [10,44]. Decades of research in SDT have shown that higher satisfaction of the three basic psychological needs, and most specifically the need for autonomy, is correlated with high quality motivation, perseverance in school and overall academic success, both in elementary, high school and post-secondary students [10]. Students with higher autonomy also have been shown to feel readier to face the inherent challenges of their academic curriculum, such as, for example, the transition from elementary to high school, while showing increased psychosocial adaptive skills, when compared to their less self-determined counterparts [45,46].

As such, interventions that aim to foster greater basic psychological needs satisfaction and decrease mental health issues are of special relevance in school-based settings. However, few published studies have attempted to experimentally manipulate basic psychological need satisfaction, especially in children. The available literature on interventions aimed at increasing need satisfaction has either targeted physical education or overall sports practice with the aim of increasing motivation [47] or parenting skills in the context of the home [48]. Results from this literature shows that when teachers or parents are trained to be more autonomy-supportive, children report higher basic psychological need satisfaction and overall well-being. Yet, authors have suggested that in regular school settings, P4C may foster greater basic psychological needs satisfaction in children [49]. This being said, in regular school settings, the available research on basic psychological need satisfaction interventions remains scarce.

## 2. Materials and Methods

### 2.1. Present Study

The goal of the present study was thus to evaluate the impact of a P4C intervention, used as an adaptation of existential therapy for children, on positive and negative indicators of mental health in elementary school children. Specifically, the impact of the intervention was evaluated pre-to-post intervention on basic psychological need satisfaction (autonomy, competence and relatedness), positive indicators (life satisfaction and self-esteem) and negative indicators (anxiety and depression symptoms) of mental health. To do so, a randomized cluster trial with a wait-list control group was implemented.

### 2.2. Hypotheses

Based on the available literature on the impact of P4C in children, we hypothesized that the intervention would have a positive impact on both positive and negative indicators of mental health. Specifically, we hypothesized that children from the experimental group would show pre-to-post increases in positive indicators of mental health and decreases in negative indicators of mental health. These improvements would be greater than those observed in children randomly assigned to the wait-list control condition.

### 2.3. Methods

Three classrooms of elementary school students from the 1st to the 3rd grade (*N* = 53, average age = 7–14) from an alternative elementary school in the Trois-Rivières area (Quebec, Canada) took part in this study and were randomly allocated, using a random numbers table, to an experimental (2 classrooms—1 classroom of 1st grade students and 1 classroom of 3rd grade students; *n* = 37) or a wait-list control condition (1 classroom of 1st grade students; *n* = 16). Recruitment occurred after their school psychologist attended a professional training session dispensed by the study’s first author; they then informed teachers in the school about the first author’s research team ongoing projects. Our team was then contacted by the school principal and the teachers who were interested in taking part in this study. Informed consent was obtained from all participating students and their parents, and from the teachers. Randomization occurred immediately after completion of pre-intervention measures. Students completed pre-intervention and immediately post-intervention measures with in-class paper questionnaires. A research assistant helped students in reading all items in the questionnaire package and answering any questions children had with regards to questionnaire items. There was no attrition in this study. Students randomly attributed to the wait-list condition were offered the intervention once the intervention with students from the experimental group was completed.

### 2.4. Intervention

As part of this study, a five-week P4C intervention was implemented. Each weekly session lasted approximately 60 min. Two themes were presented each week. The intervention was comprised of the following themes: (1) happiness; normal vs. not normal; (2) making mistakes; pride and shame; (3) mocking; separation; (4) sadness; death; (5) anger; identity and growing up (for further details, please refer to Table 1). All themes were selected based on their proximity with existential psychology and the assumption that they would foster greater basic psychological need satisfaction in children. A variety of existential primers were used throughout the intervention, with children being presented with short stories, posters, coming strips and images, which were taken from the children’s francophone magazine *Pomme D’Api’s* “Les petits philosophes” (in English: *Little Philosophers*) collection and book collection *Les Goûters Philo*, or short video clips from the francophone version of the BBC’s series *What’s the Big Idea?* The intervention was led by the second author of this paper (CDT), who had received extensive training on existential therapy-related themes of P4C. Supervision was offered weekly by the research team throughout the project. Treatment fidelity data was ensured in two ways: (1) during supervision sessions, where CDT provided specific details and feedback of which activities were completed during each session; and (2) through additional data collected from CDT’s journal and notes taken throughout the unfolding of the intervention. Upon study completion and with regards to both supervision sessions and CDT’s journal, treatment fidelity was set at 100% for this study.

### 2.5. Measures

The present study used children self-reports to assess their mental health using well-validated assessment tools. These tools have been found to be valid and reliable ways to measure children’s mental health, and children have been shown capable of accurately testifying for themselves [50]. Whereas final measures on children self-reports often have high positive correlations with parental and teachers measures, authors suggest avoiding seeing the adult as the best assessor of a child’s mental health [51].

#### 2.5.1. Positive Indicators of Mental Health

Participants rated how competent, autonomous and related they felt in school, by answering a 5-item scale adapted from a scale used in a previous, similar study [52,53]. Children were asked to rate their agreement to items such as “In school … I feel free to express my ideas” (autonomy; 1 item); “I am able to reach my goals” (competence; 1 item) and “In my relationship with others, I feel appreciated” (relatedness; 3 items). Internal consistency for the total scale (α pre/post = 0.70) and for the affiliation subscale (α pre/post = 0.70/0.75) were acceptable.

Participants also completed the five item Satisfaction with Life Scale [54] and were asked to rate to which extent they agreed with items such as “I am happy with my life”. Internal consistency was excellent for this scale (α pre/post = 0.88/0.91).

Finally, participants completed five items from the Rosenberg Self-Esteem Scale [55] and were asked to indicate how much they agreed or disagreed with items such as “I feel like I have a good number of qualities”. Internal consistency was good to acceptable for this scale (α pre/post = 0.82/0.74).

#### 2.5.2. Negative Indicators of Mental Health

Participants also completed selected items from the anxiety (2 items, e.g., “I worry about little things”) and depression (5 items, e.g., “Nothing ever goes right for me”) subscales of the Behavior Assessment Scale for Children (BASC II) [56]. Internal consistency was acceptable for both subscales (α depression pre/post = 0.63/0.85; α anxiety pre/post = 0.55/0.68).

### 2.6. Data Analysis

Based on previous work suggesting the use of ANCOVAs to increase statistical power in randomized controlled trials [57], hypotheses were tested using analyses of covariance (ANCOVAs) allowing for a comparison of post-intervention scores in each group, controlling for pre-intervention scores. Effect sizes were also computed in order to assess the magnitude of the observed effects.

## 3. Results

Preliminary analyses using independent t-tests did not show any group differences at pre-intervention on positive and negative indicators of mental health. Detailed results can be found below and in Table 2 and Table 3.

### 3.1. Positive Indicators of Mental Health

ANCOVAs revealed a significant effect of group condition on levels of autonomy (F (1, 50) = 5.47, *p* = 0.02, partial η2 = 0.10), after controlling for baseline levels. Participants in the experimental group showed greater scores in autonomy at post-test (M_post_, adjusted for baseline = 4.08), when compared to participants in the control group (M_post_, adjusted for baseline = 3.5). Specifically, participants from the experimental group showed an increase in scores from pre-intervention (M_pre_ = 3.59) to post-intervention (M_post_ = 3.84), whereas participants from the control group showed a decrease in scores from pre-intervention (M_pre_ = 3.31) to post-intervention (M_post_ = 2.81). We found no significant effect of group condition for levels of competence, relatedness, or total need satisfaction (please refer to Table 3).

ANCOVAs did not reveal a significant effect of group condition on levels of self-esteem and life satisfaction, after controlling for baseline levels (please refer to Table 3).

### 3.2. Negative Indicators of Mental Health

ANCOVAs revealed a significant effect of group condition on levels of anxiety (F (1, 39) = 7.35, *p* = 0.01, partial η2 = 0.16), after controlling for baseline levels. Participants in the experimental group showed lower scores in anxiety at post-test (M_post_, adjusted for baseline = 2.09), when compared to participants in the control group (M_post_, adjusted for baseline = 3.43). Specifically, participants from the experimental group showed a decrease in scores from pre-intervention (M_pre_ = 2.70) to post-intervention (M_post_ = 2.10), whereas participants from the control group showed an increase in scores from pre-intervention (M_pre_ = 2.67) to post-intervention (M_post_ = 3.42). We found no significant effect of group condition for depression (please refer to Table 3).

## 4. Discussion

The goal of this study was to evaluate the impact of a P4C intervention on basic psychological need satisfaction and mental health, using an experimental design with a wait-list control group. Results from this study partially support our hypotheses, as participants from the experimental group reported higher scores in the basic psychological need for autonomy and lower scores in symptoms of anxiety. These improvements were not found in participants from the control group, indicating the P4C intervention was more useful than the simple passage of time in this sample. However, results did not indicate an effect of the P4C intervention on self-esteem, life satisfaction or depression.

As stated in the introduction, one purpose of the present study was to provide empirical documentation of the impact of P4C on basic psychological need satisfaction and mental health in elementary school students. Indeed, there is a paucity of empirical data, based on experimental designs, supporting theoretical claims on the impacts of P4C in school settings. Past qualitative research from the educational and philosophical worlds has suggested P4C has benefits in the social, emotional and cognitive spheres of development [16,22,25]. Results from quasi-experimental studies have also reported benefits for special education students with regards to behavioral and emotional regulation skills [58,59]. It has also been further argued that the practice of P4C and its inherent development of critical thinking skills can help students gain a greater sense of autonomy in more controlling contexts such as special education classrooms. Similarly, although students from this study were in regular classrooms, our results support this outcome, as is shown by significant increases in autonomy scores from pre-to-post intervention in participants from the experimental group. However, given statistical and methodological limitations of this study detailed below, caution is warranted in generalizing findings from this study to larger populations.

However, results did not show a significant impact of the P4C intervention on levels of competence or relatedness. This may be due to the nature of the intervention itself, which, through its chosen themes and explicit aims, did not directly target both psychological needs in comparison to autonomy. Indeed, there was no activity that had participants reflect and exchange on their sense of competence or mastery (in school, at home, in hobbies), nor on social skills or social relationships, such as friendships or relationships with family members. Although one weekly session discussed affiliation-related themes (mocking and separation), these themes were not discussed with the aims of reflecting on how participants had significant attachment figures or friends in their lives, but more with regards to (1) mocking being (or not) socially acceptable; and (2) how it feels to be separated from one’s parents during the school day, or how it feels to be separated from a loved one or family pet that is deceased. As such, relatedness and competence, as defined by SDT, were not themes that were comprised in this intervention. The main emphasis was clearly placed on autonomy. Further iterations of this intervention could incorporate activities that would aim to bring reflection on one’s sense of competence and social relationships and evaluate if, in turn, these iterations would have a significant impact on both psychological needs with elementary school students. It is also possible that the length of the intervention could explain the absence of results, as children were only exposed to P4C for a duration of 5 weeks. This being said, it remains possible these results were obtained because of a lack of statistical power, and caution is warranted in generalizing their interpretation beyond this sample. It is also possible that the effects of P4C interventions are more significant in the long term, teaching children valuable skills for the future.

One theoretical framework that can be helpful to understand results from this study resides in the literature linking concepts pertaining to self-determination theory and mindfulness. Mindfulness can be defined as “…the process by which we pay attention in a particular way, on purpose, in the present moment, and non-judgmentally” [60]. It has been posited that, as individuals become more attuned to the present moment experience through mindfulness, they also increase their emotional regulation skills, which, in turn, fosters higher basic psychological need satisfaction [61]. Mindfulness is therefore thought to facilitate an unveiling of thoughts, emotions and physical sensations which can help individuals gain a better sense of who they are, leading to more realistic appraisals of their basic psychological needs satisfaction and helping them get in touch with their values [62,63,64]. As such, we posit that participating in P4C sessions in which existential themes are brought forward has similar effects on children, as it implies pausing to reflect on a given issue, in the present moment, without distractions or barriers clouding one’s judgement, to clarify one’s thoughts about the issue at hand. Indeed, the inherent mindful qualities of P4C may explain, at least in part, why these activities had a positive impact on autonomy in participants from the experimental group. Future studies can attempt to demonstrate this by measuring the impact of P4C on mindfulness skills directly.

The P4C intervention used in this study also had a positive impact on mental health, as shown by significant decreases in anxiety scores of participants from the experimental group. It should be noted that P4C was not initially developed as an intervention to improve negative indicators of mental health. Nevertheless, one possible way to understand P4C’s impact on anxiety is through the lens of existential therapy. Indeed, “existential psychotherapy […] further encourages clients to search for a new and increased awareness of what matters in the present. This awareness is intended to enable clients to achieve a new freedom and responsibility to act” [34] (p. 4). Although empirical data on the impact of existential therapy in youth remains scarce, its effect on improving mental health (such as, for example, reducing psychopathology and increasing meaning in life) has been documented with adults [40]. Indeed, existential therapy is structured to assist clients in noticing, acknowledging, expressing and managing psychological distress, which encompasses anxiety and depression symptoms [38,39]. Some forms of existential therapy, such as experiential-existential therapy, also place a strong emphasis on clients facing their existential thoughts and processes [65]. It is our understanding that P4C may serve similar aims with children, as these activities imply that children are faced with existential themes and are to actively think and formulate their own thoughts about these themes. As such, P4C, in the context of this study, is best understood as a means to meet an end, as it provides an already existing tool that is adapted to youth and that allows to explore themes that pertain to existential therapy with a younger population. As such, our results tend to indicate that P4C can indeed provide adequate activities and themes to allow for a useful adaptation of existential therapy for youth.

Our results did not indicate a significant impact of the intervention on depression. However, students from this sample were from a non-clinical population and baseline levels of depression for the experimental group were low and thus did not provide for much room for improvement. However, descriptive statistics show a non-significant decrease in depression scores in the experimental group, whereas participants from the control group report a slight increase in their pre-to-post scores. Finally, our results did not show a significant impact of the intervention on positive indicators of mental health. Indeed, self-esteem and life satisfaction scores did not significantly differ from pre-to-post intervention. With regards to self-esteem, given its strong ties to the basic psychological need for competence, for similar reasons as those outlined above, it is possible that themes that were explored in the P4C activities did not tap directly or specifically self-esteem as a core concept. This could be reflected in the absence of significant results. Regarding life satisfaction, we would have expected an increase in scores given the intervention’s positive impact on autonomy scores. Past research in SDT has shown that an increase in autonomy and in basic psychological needs overall typically leads to increases in well-being [66]. However, it is possible this increase does not occur immediately at post-intervention, but rather through a gradual process over time. The absence of longitudinal data does not allow us to verify this claim, but we strongly suggest that future work addresses this issue to clarify the potential impact of P4C on life satisfaction and overall if potential benefits on autonomy and mental health are maintained over time.

From a feasibility standpoint, results from this study, and anecdotal evidence collected from teachers taking part in this project and from the session leader, show the intervention was positively received and enjoyed by students and teachers alike. Themes were understood by the participants, who actively participated in the P4C activities. Visual and video content was preferred to stories and comic strips, as children could easily and rapidly understand the existential themes that were presented to them. When stories were used, CDT reported the need to often reword the content of the story and remind students of the theme during the discussion. In such instances, participants did not share nor dialogue as much, which made for less lively conversations. It is possible that the use of philosophically oriented stories may be more adequate for older elementary school children. Overall, however, the project was well received by children and the school and thus, shows good acceptability and feasibility.

### Strengths and Limitations, and Suggestions for Future Studies

This study counts notable strengths. First and foremost, the use of an experimental design with a wait-list control provided us with rigorous and valid results. Indeed, participants from all three classrooms were equivalent at baseline and were randomly allocated to each condition. Furthermore, there was no attrition in this study, which strengthens the validity of our results. As such, the observed changes were not due to changes in the sample, but rather to changes in pre-to-post scores in participants themselves. Finally, this study provides empirical data showing the promising impact of P4C as a useful tool to implement in elementary school classrooms to foster autonomy and to improve mental health from the rigorous standpoint of an experimental, randomized cluster trial. Results further show support to the validity and relevance of using P4C as an adaptation of existential therapy in youth. Further research with larger sample sizes and longitudinal data is warranted to establish the potential of such an intervention with children.

Despite these strengths, this study also has limitations. First, changes in autonomy were only measured with a single item, providing a limited evaluation of pre-to-post changes in autonomy in our sample. Although we had initially planned to administer a questionnaire comprised of three items related to autonomy to evaluate pre-to-post changes in our participants, it quickly became obvious during the pre-intervention completion of questionnaires that first grade students did not understand two of the three selected items pertaining to autonomy, with led us to the decision of keeping only one item in our final design. Indeed, given the cognitive development of first graders, it is possible that autonomy is a concept that is more difficult to grasp and understand, which leads to difficulty in trying to evaluate it in younger populations. Future work could aim to present younger students with adapted items that are easier to understand. Developing observational methods to evaluate autonomy in children could also represent an interesting option. Overall, the small number of items that were administered could reduce the sensitivity of our measures, which may also have impacted our internal validity coefficients, some of which were poor, whereas others were acceptable to good. Perhaps new significant pre-to-post changes would be detected with a larger number of administered items. Furthermore, some biases of self-assessment may be present such as social desirability and must be taken into consideration when interpreting the results. Second, the lack of longitudinal data collection prevents us from evaluating if the observed effects of the P4C intervention could have been maintained once the intervention ended and only shed light on immediate post-intervention effects of P4C on autonomy and mental health. Third, as the control wait-list condition was only comprised of 16 1st grade students, whereas the experimental condition included 37 students across 1st and 3rd grade, it is possible there might have been extraneous factors (e.g., developmental, grade level/classroom or teacher level differences) that could have also resulted in intervention effects. However, no other programs/teaching techniques occurred in the classrooms that took part in this project. Nonetheless, attention to some of these factors and possible limitations is warranted in analyzing the results of this study. Finally, increasing the sample size would also help to ensure meeting minimal requirements for adequate statistical power. As such, given this final sample size fell short of a few participants to meet such requirements, it is possible that we were not able to detect significant pre-to-post changes in participant scores and across conditions for some variables, increasing the risk of making a Type II error.

Given the fact that P4C activities are typically led by teachers or completed in their presence during regular class time, further studies could aim to incorporate observational methods to evaluate pre-to-post changes in autonomy supportive behaviours [67]. Indeed, teacher autonomy support has been shown to increase students’ engagement, motivation and overall basic psychological need satisfaction [68,69]. As such, implementing a multi-method approach in which observational and self-reported measures are combined would increase the scope of variables being evaluated and strengthen future studies’ designs. Although the pragmatic approach of P4C perceives the object of the philosophical reflections (i.e., the themes discussed) to be less relevant than the process of reflection itself, future studies should explore the direct effect of each philosophical theme on different outcomes. It could be an interesting line of investigation in the future to assess the correlations between the object of the discussion and the immediate effects on the children.

Finally, in line with what has been discussed above, combining future P4C interventions with a mindfulness meditation component could potentially increase the observed benefits on basic psychological need satisfaction and mental health in elementary school children. Although preliminary research on the impact of mindfulness-based interventions on basic psychological needs satisfaction in children has shown somewhat conflicting results with regards to its impact on need satisfaction in elementary school students [52,70,71], a combined P4C and mindfulness intervention could perhaps enhance the observed effects on autonomy specifically and mental health. Indeed, as mindfulness meditation helps children become more aware of their values through means of increased awareness, P4C activities facilitate active thinking about—and subsequent identification of—personal values. As such, we hypothesize that this would lead to more important increases in autonomy and mental health in youth than either intervention taken separately. Further studies could explore the impact of such a combined intervention and contrast this impact to both P4C and mindfulness meditation separately to evaluate the added value of such a combination. At any rate, comparing the effectiveness of P4C to another intervention within a randomized controlled trial with an active control condition would improve on the study design.

## 5. Conclusions

Overall, results from this study show that P4C may be a promising intervention to foster greater autonomy in elementary school children, while also improving their mental health. Further research is warranted to document the benefits of P4C with larger sample sizes and an active control condition.

## Figures and Tables

**Table 1:** Philosophy for children intervention.

Week	Themes	Type of Primer Used
1	Happiness Normal vs. not normal	Poster Short story
2	Making mistakes Pride and shame	Poster Short story
3	Mocking Separation	Poster and comic strip Comic strip
4	Sadness Death	Video clip Video clip
5	Anger Identity and growing up	Video clip Video clip

**Table 2:** Means and standard deviations for positive and negative indicators of mental health.

Experimental Group				
Dependent Variable	Pre-Test (SD)	Post-Test (SD)	Pre-Test (SD)	Post-Test (SD)
Positive indicators				
Autonomy	3.31 (1.66)	2.81 (1.22)	3.59 (1.54)	3.84 (1.48)
Competence	6.00 (1.36)	5.57 (1.45)	6.26 (1.19)	5.69 (1.47)
Relatedness	2.77 (1.27)	3.38 (1.28)	3.37 (1.23)	3.64 (1.21)
Life satisfaction	10.80 (2.57)	11.60 (0.84)	11.42 (1.79)	10.08 (3.52)
Self-esteem	12.75 (3.33)	13.75 (2.45)	13.61 (3.45)	12.54 (2.86)
Negative indicators				
Anxiety	2.67 (1.82)	3.42 (1.56)	2.70 (1.84)	2.10 (1.73)
Depression	5.00 (2.00)	5.43 (3.41)	3.16 (2.83)	2.74 (2.62)

**Table 3:** Results of ANCOVA for positive and negative indicators of mental health.

Variable	df	F	*p*	Partial η^2^	Statistical Power
Positive indicators					
Autonomy	1	5.467	**0.023**	0.099	0.69
Competence	1	0.002	0.966	0.000	0.05
Relatedness	1	0.181	0.672	0.004	0.07
Total need satisfaction	1	2.258	0.140	0.047	0.38
Life satisfaction	1	3.358	0.076	0.092	0.67
Self-esteem	1	3.237	0.081	0.085	0.62
Negative indicators					
Anxiety	1	7.353	**0.010**	0.159	0.90
Depression	1	2.708	0.109	0.072	0.54

Note: Bold: *p* < 0.05.

## Data Availability

The data presented in this study are available on request from the corresponding author. The data are not publicly available due to ethical restrictions.
